# Effects of Oxytocin Receptor Agonism on Acquisition and Expression of Pair Bonding in Male Prairie Voles

**DOI:** 10.21203/rs.3.rs-4351761/v1

**Published:** 2024-05-15

**Authors:** Andrey Ryabinin, Michael Johnson, Jonathan Zweig, Yangmiao Zhang, Louis Nunez, Olga Ryabinina, Marcel Hibert

**Affiliations:** Oregon Health & Science University; Oregon Health & Science University; Oregon Health & Science University; Oregon Health & Science University; Oregon Health & Science University; Oregon Health & Science University; CNRS / Université de Strasbourg

## Abstract

There is much interest in targeting the activity in the oxytocin system to regulate social bonding. However, studies with exogenous administration of oxytocin face the caveats of its low stability, poor brain permeability and insufficient receptor specificity. The use of a small-molecule oxytocin receptor-specific agonist could overcome these caveats. Prior to testing the potential effects of a brain-penetrant oxytocin receptor agonist in clinical settings, it is important to assess how such an agonist would affect social bonds in animal models. The facultatively monogamous prairie voles (*Microtus ochrogaster*), capable of forming long-term social attachments between adult individuals, are an ideal rodent model for such testing. Therefore, in a series of experiments we investigated the effects of the recently developed oxytocin receptor-specific agonist LIT-001 on the acquisition and expression of partner preference, a well-established model of pair bonding, in prairie voles. LIT-001 (10 mg/kg, intraperitoneal), as expected, facilitated the acquisition of partner preference when administered prior to a 4-hour cohabitation. In contrast, while animals injected with vehicle after the 4-hour cohabitation exhibited significant partner preference, animals that were injected with LIT-001 did not show such partner preference. This result suggests that OXTR activation during expression of pair bonding can inhibit partner preference. The difference in effects of LIT-001 on acquisition versus expression was not due to basal differences in partner preference between the experiments, as LIT-001 had no significant effects on expression of partner preference if administered following a shorter (2 hour-long) cohabitation. Instead, this difference agrees with the hypothesis that the activation of oxytocin receptors acts as a signal of presence of a social partner. Our results indicate that the effects of pharmacological activation of oxytocin receptors crucially depend on the phase of social attachments.

## Introduction

Social relationships and attachments are central to the well-being of humans. Disruption of such relationships can have devastating consequences. More specifically, lack of social interaction contributes to increases in anxiety and depression, excessive substance use, and self-harm [1, 2]. Therefore, there is a great interest in understanding the mechanisms regulating social relationships and manipulation of their various components to improve the formation and maintenance of such relationships.

Prairie voles (*Microtus ochrogaster*) have proven to be an essential model for understanding mechanisms regulating social attachment behavior [3–6]. It is well demonstrated that in the wild prairie voles can establish and maintain long-term attachments with the specific individuals of the opposite sex, aka pair bonds [7–9]. Therefore, similarly to humans, they are considered facultatively monogamous [10–12]. A pair bond is defined by a long-term emotional association between mating partners. Pair bonds are behaviorally reflected in selective affiliation, selective aggression against unfamiliar conspecifics (aka strangers), preferential (but not exclusive) mating and high level of distress upon separation [13 and references within]. The process of forming a pair bond is defined as pair bonding and involves development of these behaviors.

In the laboratory, selective affiliation can be detected using the partner preference test (PPT), in which previously cohabitating individual animals spend preferential time in a side-by-side contact, aka huddling, with their partner versus a novel opposite-sex individual [14, 15]. Partner preference is not observed in non-monogamous species and, therefore, often serves as the laboratory proxy for the process of pair bonding [6, 9]. Selective affiliation, or partner preference, is one of the early expressions of initial pair bonding, as it can be observed as early as after just one hour of a male-female exposure in prairie voles [16–18], although many laboratories use longer periods of cohabitation [6, 15]. These observations imply that this cohabitation leads to acquisition of a memory of the partner animal. This memory is then later expressed in the preferential huddling with this animal over a stranger in the PPT.

The role of central oxytocin (OXT) signaling in the regulation of pair bonding was first discovered in prairie voles [8, 19]. OXT is an evolutionarily conserved nonapeptide produced by magnocellular and parvocellular neurons in the paraventricular nucleus of the hypothalamus (PVN), the supraoptic nucleus of the hypothalamus and, to a lesser extent, in the accessory nucleus of the hypothalamus [20–25]. OXT is a vital signaling molecule in the neuroendocrine system, important for the regulation of reproduction, childbirth, and social behaviors [20, 26, 27]. Facultatively monogamous species of voles, including prairie voles, have a uniquely different central oxytocin receptor (OXTR) distribution when compared to non-monogamous species of voles [28–30]. Accordingly, central administration of OXT and its closely related peptide arginine vasopressin (AVP) can facilitate the development of pair bonding in both sexes, although AVP in males requires lower doses to produce these effects than OXT [16, 19, 31]. Administration of OXTR antagonist into the nucleus accumbens of male prairie voles prior and during prolonged cohabitation inhibited partner preference [32]. Viral manipulations of gene expression have confirmed the promoting role of OXTR on pair bonding in female prairie voles [33, 34]. Meanwhile, in humans, intranasal OXT treatment improves reported social trust and social altruism, whereas variations of single nucleotide polymorphisms in the OXTR gene are related to pair bonding and other social behaviors [35–37]. A substantial number of clinical trials have reported OXT’s therapeutic efficacy in the treatment of psychiatric conditions such as autism spectrum disorder (ASD), depression, and addiction [38–42]. Although OXT’s mechanistic role in treating neuropsychiatric conditions is still far from fully understood, it is thought to ameliorate symptoms by engaging circuitry promoting social cues and reward [for recent review see 43]. Ergo, the OXT system is intimately coupled with social bonding behaviors in both prairie voles and humans.

Despite this accumulated evidence, however, recent studies using CRISPR to knockout OXTRs in prairie voles, revealed that these receptors are not entirely necessary for pair bonding, and that prolonged cohabitation can induce pair bonding in the absence of OXTRs [44]. Such findings indicate redundancy in the mechanisms regulating social attachment and suggest that OXT signaling acts to enhance rather than directly precipitate social bonding. While many studies have relied on the exogenous application of OXT, there is much controversy about how efficient OXT is when administered via any route [45–47] as OXT has low brain penetrance and can also act on other central receptors, specifically AVP V1a and V1b receptor subtypes [45, 48–51]. OXTR-specific agonists have been used to test the roles of these receptors in neuronal activity associated with pair bonding [52, 53]. However, the peptide-based nature of these agonists did not overcome the problem with brain penetration in behavioral studies. A way to overcome these caveats is the application of small molecule agonists. One such agonist, LIT-001 (LIT), has been recently developed. Unlike OXT, LIT readily crosses the blood brain barrier, and is highly specific for OXTR [54]. Thus far, there have been limited studies using LIT. In a genetic mouse model of ASD, LIT has been demonstrated to restore social interactions [54]. In rats, LIT has been shown to have anti-nociceptive effects [55]. In socially housed male prairie voles LIT decreased voluntary alcohol consumption [56]. However, how such an OXTR-specific, blood brain barrier-penetrating agonist could affect social attachment in prairie voles has not been evaluated.

The ability to test the specific role of OXTR innervation in pair bonding using LIT in prairie voles opens many previously unavailable opportunities. In particular, it allows for the first time the possibility of evaluating whether activation of OXTR would differentially contribute to different phases of pair bonding. Thus, administration of LIT before cohabitation between the future partner animals allows testing effects of OXTR activation on acquisition of pair bonding. On the other hand, administration of LIT after cohabitation allows testing effects of OXTR activation on expression of pair bonding. Such testing could assess various behavioral attributes of a pair bond, such as selective affiliation, selective aggression, preferential mating, or distress upon separation. Previous pharmacological studies assessed effects of administration of OXT or OXT antagonists on acquisition of pair bonding and revealed facilitating effects, although whether these facilitating effects were selective to OXTR in males has been debated [9, 10, 16, 19, 57]. Viral manipulations of gene expression did not allow to distinguish the role of OXTR in acquisition versus expression of pair bonding [33, 34]. Studies demonstrating that administration of OXT inhibits indices of separation-induced distress suggest OXTR’s role in expression of pair bonding [58–60], but can be difficult to interpret because OXT also has anxiolytic effects in non-social paradigms [61, 62]. No other studies on the role of OXTR in expression of pair bonding have been performed. To address this gap in knowledge, we compared the effects of LIT on partner preference in male prairie voles by either injecting it after cohabitation and prior to PPT (testing its effects on expression of pair bonding) or prior to the cohabitation (testing its effects on acquisition of pair bonding). Our results demonstrate differential effects of OXTR activation at these two phases on pair bonding experiments.

## Methods

### Animals

Adult male and female prairie voles from our laboratory’s colony at Oregon Health & Science University (OHSU) were used in these experiments. All experiments were approved by the Institutional Animal Care and Use Committee at OHSU, Portland, OR, USA and conducted in accordance with the National Institutes of Health (NIH) Guidelines for the Care and Use of Laboratory Animals. Prairie voles were weaned at 21 days old and housed in same sex groups in standard individually ventilated cages (27 × 27 × 13cm) with cotton nestlets, cellulose bedding and Crink-l’nest bags (The Andersons, Maumee OH), white FlexiChew Bone Dog Chew Toys (Nylabone, Neptune City, NJ) and a wooden cube for enrichment. Animals had free access to water, rabbit chow (Laboratory Rabbit Diet High Fiber, 5326; LabDiet, St.Louis, MO), Timothy hay (Hand-Selected Timothy Grass; Standlee Premium Products, Kimberly, ID) and rodent diet 5LOD (LabDiet). Female and male animals were housed in separate rooms prior to experimental cohabitation to prevent induction of ovulation as female prairie voles do not display estrus cycles unless directly exposed to males. To ensure a relative genetic diversity of our colony, we maintained at least 12 breeding pairs, and periodically imported prairie voles from other institutions. Breeding pairs were set up with animals as unrelated as possible. Animal rooms were kept on a 14:10 light:dark cycle with lights on at 6AM. We used males rather than both sexes of voles as test subjects in the study because of following two reasons. First, while the importance of OXTR in pair bonding for females has been well established, several studies questioned their roles for pair bonding in males [10, 31, 57]. Second, while we were in the process of discontinuing our vole colony, we had a greater number of female than male animals, and the partner preference experiments require twice many opposite sex animals to those used as test subjects (see Partner Preference Test below). The number of animals per group, therefore, was based on previous studies reporting significant effects of treatments on partner preference in males [63, 64] and availability of animals. To achieve the required number of animals, each experiment was run in several cohorts containing as equal representation of all groups tested in the experiment as possible. Data from each cohort was combined into each single experiment that was not further replicated.

### Drugs

OXT agonist LIT-001 HCl salt was synthetized at University of Strasbourg. Aliquots of the compound were dissolved in DMSO to a concentration of 20 mg/ml and frozen at −20°C. Prior to animal treatment, aliquots were diluted in 0.9% sodium chloride saline (Hospira, Lake Forest, IL RL-4415) to 1 mg/ml. Animals were treated with 10 mg/kg LIT-001 based on previous experiments, where this dose decreased alcohol intake in male voles without affecting water intake or approaches to the water spout, indicating lack of sedative effects [56]. Vehicle treatments were 10 ml/kg of 5% DMSO in 0.9% sodium chloride saline. Only male animals were treated in these experiments and all treatments were administered as intraperitoneal (i.p.) injections. Oxytocin antagonist L-368,899 (R&D systems, Minneapolis, MN) was dissolved in DMSO to a concentration of 20 mg/ml and frozen at − 20°C. Prior to animal treatment, aliquots were diluted in 0.9% sodium chloride saline (Hospira, Lake Forest, IL) to 1 mg/ml. Animals were treated with L-368,899 at 10 mg/kg, i.p.

### Cohabitation

During cohabitation, sexually naive partner male and partner female were moved to a new cage (27 × 27 × 13cm) containing fresh bedding, water, chow, and enrichment supplies and allowed to interact undisturbed for the time required for experimental parameters. Males used as test subjects in the study were between 13 and 24 weeks old. Females used as stimulus animals in the study were between 10 and 27 weeks old. Males and females used in experiments were selected not randomly but based on being from different litters and unfamiliar to each other.

There is variability between laboratories in the duration of cohabitation required to establish partner preference. Our preliminary pilot tests indicated that 4 hour of cohabitation was sufficient to produce partner preference. This interval is shorter than the cohabitation interval used by many laboratories [15]. However, even 1-hour intervals have been successfully used to study treatments enhancing partner preference [10, 16, 17, 57]. Therefore, we used 4 and 2 hours of cohabitation intervals in the current study.

In the experiment, where observations during cohabitation were required, filter tops and wire food holders were removed and replaced with a transparent plastic aerated lid. Since this necessitated the removal of food and water, cups of rodent diet 5LOD (Lab Diet, St. Louis, MO) were added to cohabitation cages with added autoclaved water. No mating was observed during the cohabitation.

### Partner Preference Test

**Figure 1:**
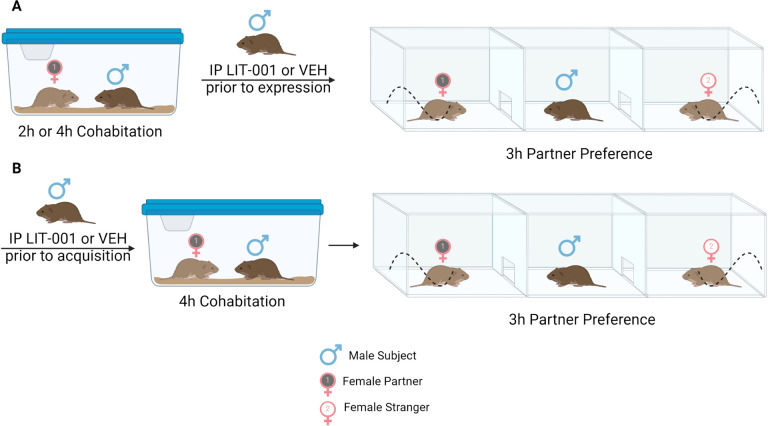
Schematic representation of experiments. A. In experiments testing effects of oxytocin agonism on expression of pair bonding (shown in Figures 2 and 4), male prairie voles were injected with LIT (10 mg/kg) or vehicle after cohabitation with a female and prior to the partner preference test. B. In the experiment testing effects of oxytocin agonism on acquisition of pair bonding (shown in Figure 3), male prairie voles were injected with LIT (10 mg/kg) or vehicle prior to cohabitation with a female. Illustration created with biorender.com.

The Partner Preference Test (PPT) is an established method for assessing pair bonding [14, 15]. Within a three chambered linear apparatus (22 × 75 × 30 cm, each sub chamber approximately 25 cm in length), a female partner vole is tethered to the end of one chamber and a same age unfamiliar female (stranger) vole is tethered to the end of the opposite chamber (Fig. 1). The partner male is introduced to the middle chamber and can freely explore the apparatus for three hours. Huddling time between the male and the female animals was measured as the duration of persistent, non-coincidental side-by-side physical contact. The preferential huddling was calculated by subtracting huddling time with the stranger from huddling time with partner. Also measured was the time the male animal spent in any single chamber and number of aggressive events (lunges and chases of one animal by another). No mating occurred during the tests. Behavioral scoring was done at 1:1 speed using VLC Media Player (Boston, MA, USA) and JWatcher V1.0 (http://www.jwatcher.ucla.edu). The investigator performing the scoring was blinded to the group assignment of animals.

### Statistical Analyses

Because some values for huddling time and number of aggressive events equaled zero, the distribution of measurements was not normal for several tests. Therefore, non-parametric tests were used for statistical analyses. The existence of significant differences between behavioral measures was first determined by a Kruskal-Wallis test. Significance of differences between specific a *priori*-determined measures was then analyzed by post-hoc Mann-Whitney U tests. Preferential huddling was analyzed for significant difference from zero using the one-sample Wilcoxon signed rank test. All statistical analyses were performed using SPSS (IMB Corp. Version 29.0, Armonk, NY). All graphs were built using GraphPad Prism (GraphPad Software, Version 10.2, Boston MA). Results below only indicate P values of relevant tests. Graphs depict mean plus minus standard error of the mean (SEM), as well as values of each individual data points. Supplemental Table reports all statistical analyses in order of their appearance in the text.

## Results

### Effects of LIT on expression of pair bonding.

**Figure 2:**
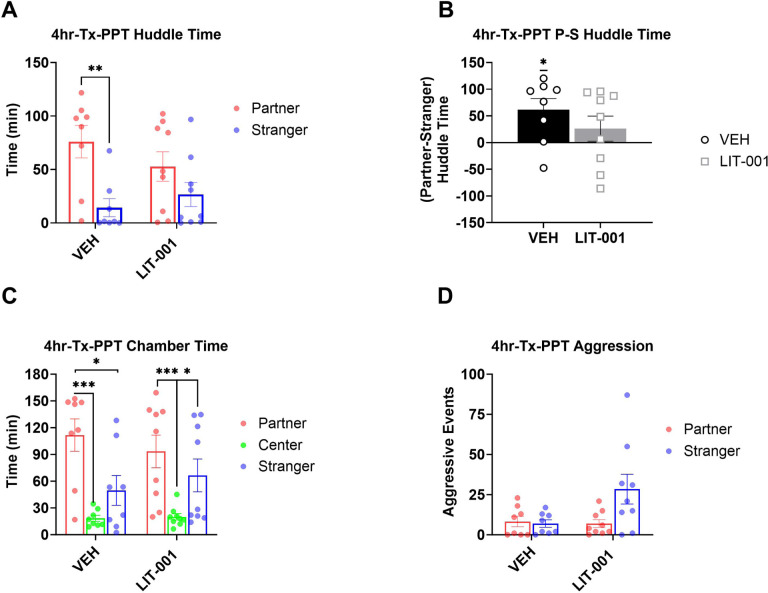
Analysis of male prairie voles that received LIT (10 mg/kg, n=9) or vehicle (n=8) after a 4-hour cohabitation and prior to a 3-hour partner preference test. A. Total huddling time. B. Preferential huddling. C. Time spent in respective chambers by male prairie voles. D. Aggressive events. All bars reflect mean ± SEM. *, p<0.05; **, p<0.01; ***, p<0.001, A, C, and D Kruskal-Wallis test followed by a post-hoc Mann-Whitney analysis, B One-Sample Wilcoxon Signed Rank Test.

To investigate the effects of systemic OXTR agonism on expression of pair bonding, we injected male prairie voles with LIT (10 mg/kg, i.p.) or vehicle following a 4-hour cohabitation and immediately prior to a PPT (Fig. 1A). Males that received vehicle injection prior to the PPT spent significantly more time huddling with partner versus stranger voles (Fig. 2A, Kruskal-Wallis P = 0.009, Mann-Whitney P = 0.003), indicative of the establishment of partner preference. In contrast, there was no difference in the amount of time male voles injected with LIT huddled with the partner compared to the stranger animal (Fig. 2A, Mann-Whitney P = 0.19). Correspondingly, the preferential huddling was significantly different from zero in vehicle-injected voles (Fig. 2B, Wilcoxon P = 0.036), but not in LIT-injected voles (Fig. 2B, Wilcoxon P = 0.21). These findings suggested that OXTR agonism interfered with the expression of pair bonding in male prairie voles.

Previous *in vitro* experiments have demonstrated high specificity of LIT as an agonist of OXTR [54, 55]. To confirm LIT’s specificity towards this receptor *in vivo* in prairie voles, we evaluated partner preference following co-administration of LIT with OXTR antagonist L-368899 (10 mg/kg, i.p.) prior to PPT in a small group of male prairie voles run in parallel to the experiment above (Figure S1). The small number of animals did not allow us to perform the Mann-Whitney post-hoc tests on the huddling times (Kruskal-Wallis P = 0.0006 for both groups) or Wilcoxon tests for this experiment (see supplementary statistics table). However, the huddling times and the preferential huddling for animals injected with L-368899 were nominally identical to those in vehicle-injected voles in the experiment above. The partner huddling times and preferential huddling for animals co-injected with LIT and L-368899 were even higher than in vehicle-injected voles (Figure S1A,B; Fig. 2A,B). These data suggested that OXTR antagonist counter-acted the possible inhibitory effects of LIT on expression of pair bonding and agree with the OXTR-specificity of LIT’s inhibitory effect on partner preference.

While LIT has been shown to effectively penetrate the rodent brain within 30 minutes following administration and is mostly eliminated from the brain at 5 hours in rats, the dynamics of its actions in vole brain is not known [55]. To assess whether a potentially short half-life of LIT would prevent our ability to observe stronger effects of LIT occurring at shorter intervals, we examined huddling times during the first and last half of the 3-hour PPT. Huddling time between the partner and stranger during the first 90 minutes of the PPT was statistically significant for vehicle-injected animals (Figure S2A, Kruskal-Wallis P = 0.0076, Mann-Whitney P = 0.005) but did not reach Significance for LIT-injected animals (Mann-Whitney P = 0.077). Preferential huddling was low in both groups of animals, but still significantly higher than zero in vehicle and LIT-injected voles during the first half of the PPT (Figure S2C, Wilcoxon P = 0.05 and P = 0.038 respectively). During the second half of the PPT, preferential huddling was higher than zero in vehicle injected males but not in LIT-injected males (Figure S2D, Wilcoxon P = 0.043 and P = 0.67, respectively). Thus, in contrast to the theorized short-lived effects, the inhibitory influences of LIT on preferential huddling appeared stronger during the second part of the test. Therefore, in subsequent analyses we examined behavior across the entire PPT and both halves of this test.

While huddling duration is a sensitive measure of pair bonding, additional information about animals’ affiliative behavior can be derived from analyses of times spent in chambers during the PPT [6]. In agreement with effects of LIT on pair bonding determined by huddling duration and preferential huddling, vehicle-injected males spent more time in the chamber with their partners versus stranger females (Fig. 2C, Kruskal-Wallis P = 0.0006, Mann-Whitney P = 0.028), in contrast to LIT-injected males not showing this difference (Mann-Whitney P = 0.13). Similar to the analysis of huddling, the difference between time spent in partner versus stranger chambers was statistically detectable in vehicle- but not LIT-injected voles during the first half of the PPT (Figure S2E, Kruskal-Wallis P = 0.0008, Mann-Whitney P = 0.028 and P = 0.062 respectively). In addition, times spent in the partner chamber versus the neutral center chamber were significantly longer for vehicle-injected voles for the entire 3-hour PPT (Fig. 2C, Kruskal-Wallis P = 0.0006, Mann-Whitney P = 0.0018) and for each half of the test (Figure S2E Kruskal-Wallis P = 0.0008, Mann-Whitney P = 0.0029, and Figure S2F Kruskal-Wallis P = 0.0092, Mann-Whitney P = 0.0066, respectively), whereas it was significantly higher only for the entire three hour test (Fig. 2C, Mann-Whitney P = 0.00078) and the first half of the testing period (Figure S2E, P = 0.00049) in LIT-injected animals. Thus, analysis of chamber times agreed with the inhibitory effects of LIT on expression of pair bonding as derived from the analysis of huddling times.

Selective aggression towards stranger voles can serve as an additional measure of pair bonding [5, 6, 65, 66]. The number of aggressive interactions was low and there were no statistically significant differences in the number of aggressive interactions between LIT-injected and vehicle-injected animals when analyzed across the total 3 hours of the PPT or its first 90 minutes (Figs. 2D and S2G, Kruskal-Wallis P = 0.12 and P = 0.26, respectively). However, during the last half of PPT, there were more aggressive events in encounters with the stranger versus partner in LIT-injected males (Figure S2H, Kruskal-Wallis P = 0.021), but not vehicle-injected animals. Since this aggression with strangers could be considered an apparent contradiction to the decrease in partner preference in LIT-injected males, we re-examined the instances of these aggressive encounters. This analysis revealed that all aggressive events were exclusively exhibited by the stranger females, and not by the focal male animal, a finding suggesting that LIT did not increase aggression in injected males.

### Effects of LIT on acquisition of pair bonding.

Previously, OXT has been repeatedly shown to facilitate pair bonding [9, 16, 19, 67, 68]. Yet, in experiments above OXTR agonism inhibited partner preference behavior. To test whether OXTR agonism can have different effects on expression versus acquisition of pair bonding, we administered LIT or vehicle to male prairie voles immediately prior to the 4-hour cohabitation with a female and performed PPT immediately after this cohabitation (Fig. 1B). Since we observed a tendency towards an increased number of aggressive encounters in tests of LIT-injected animals in prior experiments, huddling and aggressive events were also measured during the cohabitation of these animals.

**Figure 3:**
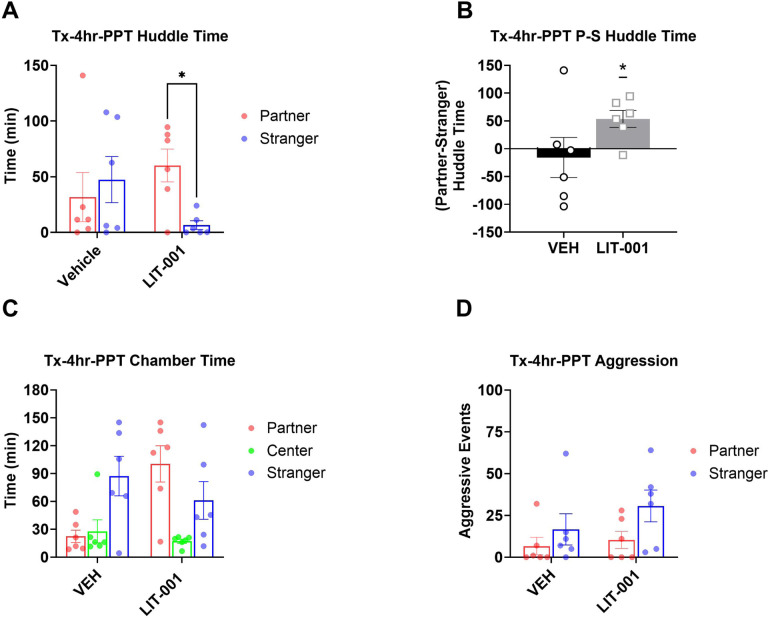
Analysis of male prairie voles that received LIT (10 mg/kg, n=6) or vehicle (n=6) prior to a 4-hour cohabitation and followed by a 3-hour partner preference test. A. Total huddling time. B. Preferential huddling. C. Time spent in respective chambers by male prairie voles. D. Aggressive events. All bars reflect mean ± SEM. *, p<0.05, A, C, and D Kruskal-Wallis test followed by a post-hoc Mann-Whitney analysis, B One-Sample Wilcoxon Signed Rank Test.

Analysis of the PPT did not reveal statistically significant differences in huddling times between partner and stranger across treatments (Fig. 3A). However, *a priori* comparisons detected significantly longer times spent huddling with partner versus stranger in LIT-injected (Fig. 3A, Kruskal-Wallis P = 0.23, Mann-Whitney P = 0.043) but not in vehicle-treated voles (Mann-Whitney P = 0.93), suggesting that LIT enhances acquisition of partner preference. In agreement, the preferential huddling was significantly different from zero in LIT-, but not vehicle-injected voles, when analyzed across the entire PPT period (Fig. 3B, Wilcoxon P = 0.046, P = 0.6, respectively). This conclusion is supported by times spent in the respective chambers. While there were no differences in times spent in the chambers in vehicle-injected animals, there was a significantly longer time spent in the partner chamber versus the center chamber during the second half of the partner preference test in LIT-injected voles (Figure S3F, Kruskal-Wallis P = 0.023, Mann-Whitney P = 0.004).

In agreement with lack of effects on aggression, there were no effects of LIT on aggressive events during the PPT (Fig. 3D) or during cohabitation (Figure S4B). Additionally, analyses of huddling time during the cohabitation did not reveal any significant effects of LIT (Figure S4A).

### Effects of LIT on partner preference after a shorter cohabitation.

**Figure 4:**
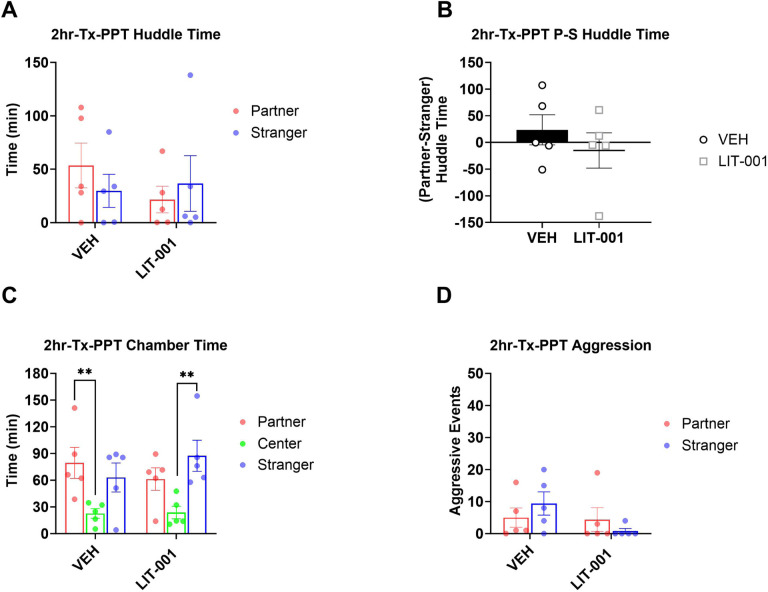
Analysis of male prairie voles that received LIT (10 mg/kg, n=5) or vehicle (n=5) after a 2-hour cohabitation and prior to a 3-hour partner preference test. A. Total huddling time. B. Preferential huddling. C. Time spent in respective chambers by male prairie voles. D. Aggressive events. All bars reflect mean ± SEM. **, p<0.01, A, C, and D Kruskal-Wallis test followed by a post-hoc Mann-Whitney analysis, B One-Sample Wilcoxon Signed Rank Test.

Vehicle-injected animals demonstrated robust partner preference in the experiment testing effects of LIT on expression of pair bonding (Fig. 2A) but did not show partner preference in the experiment testing acquisition of pair bonding (Fig. 3A). Therefore, it was theoretically possible that effects of LIT varied between the two experiments not because of differences in effects of LIT on different phases of pair bonding, but because of the initial differences in partner preference in vehicle-injected animals. To address this possibility, we tested LIT’s effects on expression of partner preference following a shorter period of cohabitation. Specifically, we administered LIT to male prairie voles after a 2-hour cohabitation, immediately before the PPT (Fig. 1A). There were no significantly differences in huddling times during PPT, and the preferential huddling was not different from zero, suggesting a lack of facilitating effects of LIT on expression of pair bonds (Fig. 4A,B). In addition, vehicle-injected voles, but not LIT-injected voles, spent more time in the partner versus center chambers (Fig. 4C, Kruskal-Wallis P = 0.019, Mann-Whitney P = 0.0079, Man-Whitney P = 0.095, respectively). Data from this experiment confirmed that LIT administered after cohabitation does not facilitate pair bonding. Taken together with the previous experiments, these results indicate that the direction of LIT’s effect (facilitating or inhibitory) is not a function of differences in the strength of partner preference but depend on the timing of its administration. Aggression episodes were rare and were not affected by LIT (Fig. 4D).

## Discussion

Our results demonstrate differential effects of oxytocin receptor agonism on expression and acquisition of pair bonding in male prairie voles. Specifically, we show for the first time that administration of an OXTR-specific agonist after cohabitation does not facilitate and possibly disrupts the expression of preferential affiliative behavior of a male animal towards its partner. In contrast, this agonist’s presence during the acquisition of pair bonding facilitates such preferential affiliative behavior. In conjunction, these two points indicate that effects of OXTR agonism on indices of social bonding are highly dependent on the social context of this treatment.

We point out that our analysis of huddling behavior was done using non-parametric tests that did not provide a statistical assessment of interactive effects between treatments (LIT versus vehicle) and stimulus animal (partner versus stranger), which would have been possible by ANOVA. Therefore, our findings of inhibitory effects of OXTR agonism on expression of pair bonding and facilitating effects of OXTR agonism on acquisition of pair bonding could be viewed as suggestive. However, the inhibitory effect on expression of pair bonding manifested itself not only in a significant difference in huddling of vehicle-injected males with their target females and lack of such significant difference in LIT-injected males (Fig. 2A), but also in a significant preferential huddling in vehicle-, but not in LIT-injected animals (Fig. 2B), and in significant difference in times spent in partner’s chamber versus stranger’s chamber in vehicle-, but not LIT-, injected voles (Fig. 2C). Therefore, we believe that this experiment provides sufficient evidence for the negative influence of OXTR agonism on the expression of pair bonding.

The inhibitory effects of OXTR stimulation on expression of pair bonding are in agreement with studies demonstrating that administration of OXT inhibits measures of distress and depression-like behaviors in socially-isolated animals, including male and female prairie voles that have been previously pair-bonded [58–60]. Moreover, administration of OXTR antagonist or viral knockdown of OXTR expression in nucleus accumbens produced depression-like behavior in male pair-bonded prairie voles after separation from their partner and even in the presence of their partner [60]. These studies suggested that activation of OXTR substitute for the social buffering effects of the presence of social partner, while blockade of OXTR activity counteracts such effects. However, interpretations of studies on effects of OXT separation-induced distress are difficult to make because OXT can also have anxiolytic effects in non-social paradigms [61, 62]. In the future, it would be beneficial to examine effects of LIT on expression of pair bonding via other indices of pair bonding besides partner preference, such as selective aggression, preferential mating, and separation distress. However, development of selective aggression and other indices of pair bonding, could require longer periods of cohabitation than employed in our study.

In fact, our study used shorter cohabitation times (4 and 2 hours) than a larger number of previous studies on pair bonding in prairie voles [14, 15]. Many laboratories use 6 hours as the shortest interval of cohabitation that does not lead to significant partner preference or other behaviors indicative of a pair bond, but allows for the observation of facilitation of pair bonding by various manipulations. These laboratories then use 24-hour or even longer intervals of cohabitation to allow studies on inhibition of pair bonding [9, 19, 69]. However, the length of cohabitation required to establish a pair bond frequently differs between laboratories. Factors contributing to this difference between laboratories are unclear, but are unlikely to involve the configuration of testing chambers [15, 70].

Many factors leading to differences in the time of cohabitation required for pair bonding remain unexplored. They could involve differences in diets and housing conditions (for example, ventilated versus non-ventilated cages). Environmental stress and resulting changes in glucocorticoid levels can enhance partner preference in male prairie voles [71, 72]. It is possible that levels of environmental stressors, due to differences in housing conditions, are different between laboratories or animal colonies. Effects of environmental conditions on pair bonding are worthy of systematic assessments. Importantly, different behaviors associated with pair bonding, including partner preference, selective aggression, preferential mating, and separation distress, could require different cohabitation times for their development. Moreover, even in this case, they would assess the process of pair bonding, but not the strength of the pair bond. The strength of the bond assumes its stability across time, which has been investigated in only a few studies [9, 69], and is deserving future experimental research. Nevertheless, a substantial number of other reports have successfully used cohabitation times as short as 1 hour to evaluate effects on pair bonding using the PPT [10, 16–18, 57, 73]. We note that these studies included experiments performed on animals only a few generations removed from wild-caught animals [10, 16]. In that sense, our observations of significant partner preference following 4 hours of cohabitation should not be considered unusual.

As expected with short cohabitation intervals, we did not see development of selective aggression in the male subjects in this study. In fact, the number of aggressive events in males were overall very low. For example, all aggressive events in the experiment testing effects of LIT on expression of pair bonding (Fig. 2D) were produced by stranger females and not by the LIT-injected focal male voles. In agreement with this, there was no difference in aggressive events during PPT when LIT was administered prior to cohabitation (Fig. 3D) and there was a tendency of decreased, rather than increased, aggressive encounters during cohabitation (Figure S4B). Therefore, the overall effect of LIT on pair bonding cannot be explained by its effects on aggression. Instead, we hypothesize that the specific activation of OXTR signals the presence of a social partner. Specifically, when LIT is administered in males that have established selective affiliation, this manipulation substitutes for the partner-induced activation of OXTRs resulting in indiscriminate affiliative behavior towards both partner and stranger female voles.

As mentioned previously, a facilitation of acquisition of pair bonding by OXTR agonism was an effect expected from the previous literature [9, 10, 16, 19, 57]. The ability to observe the facilitating effects of LIT were aided by the fact that partner preference in vehicle-injected voles was lower in the experiment testing the effects on acquisition (Fig. 2A) than in the experiment testing expression of pair bonding (Fig. 3A). This less robust partner preference in vehicle-injected voles was likely due to the fact that the cohabitation procedure was modified from the first experiment to allow observing animals (see Methods). This modification could have distracted the animals and contributed to weaker pair bonding. However, the difference in the strength of partner preference is not a factor contributing to opposite directions of LIT’s effects on acquisition versus expression of pair bonding because we did not observe any facilitating effects of this treatment on expression of pair bonding in the experiment with two hours of cohabitation (Fig. 4A).

The facilitating effects of LIT on the acquisition phase of pair bonding in our study manifested themselves in a significant difference in huddling time with partner versus stranger animals and in a significant preferential huddling in LIT-injected, but not vehicle-injected test animals during the entire PPT (Fig. 3A,B). These effects are also in agreement with the observed significant difference in time spent in partner’s chamber in LIT-injected voles, but not in the vehicle-injected voles in the second half of the PPT (Figure S3F). While these comparisons were also done using non-parametric tests, they are in overall agreement with publications showing that central administration of OXT can promote acquisition of pair bonding [9, 16, 19]. An early study demonstrated that acquisition of pair bonding in male prairie voles, measured by partner preference and selective aggression against strangers, was inhibited by central administration of a AVP receptor, but not an OXTR, antagonist, and that central administration of AVP, but not OXT, promoted acquisition of pair bonding [31]. Two subsequent studies in female prairie voles showed that central administration of OXT promoted acquisition of pair bonding, measured by partner preference, and that OXTR antagonist, but not AVP antagonist, inhibited this behavior [19, 68]. These findings gave rise to an early hypothesis that OXTR regulates pair bonding in females, whereas AVP receptors regulate pair bonding in males. This hypothesis was addressed by a direct comparison between males and females showing that central administration of either OXT or AVP can promote acquisition of pair bonding in both sexes, but that this behavior in males is sensitive to lower doses of AVP than OXT [16]. The same comparative study showed that the facilitating effects of OXT were attenuated by either administration of an OXT or an AVP receptor antagonist. Thus, both receptors are involved in facilitating effects of OXTR on acquisition of pair bonding with a potential additional reliance on AVP receptors in males. Subsequent studies using peripheral administration of peptides showed that OXT promoted acquisition of pair bonding in female, but not male, voles suggesting lesser reliance of this behavior on OXTR in males [10, 57]. On the other hand, administration of an OXTR antagonist into the nucleus accumbens of male prairie voles or into the caudate putamen of female prairie voles prior and during cohabitation inhibited subsequent partner preference [32, 74]. Experiments using viral overexpression and RNA interference in the nucleus accumbens definitively indicating the role of OXTR in formation of pair bonding did not clarify its role in male prairie voles, as they were performed only in females [34, 75, 76]. Thus, while the facilitating role of OXT in acquisition of pair bonding is well established, the specific role of OXTR in this behavior in males is not completely delineated. Our finding of facilitating effects of LIT on acquisition of pair bonding indicates the specific importance of OXTR for this behavior in males. This finding does not mean that AVP receptors are not involved in this behavior, only that activation of OXTRs is sufficient to facilitate acquisition of pair bonding.

Our need to rely on non-parametric tests in statistical analyses of behavior was driven by the lack of normal distribution of our data. This lack of normality is primarily due to presence of a substantial number of zero values. This distribution is expected when using short cohabitation intervals which may lead to low measures of partner preference, particularly huddling time, that can also be disrupted by treatments. As in other studies using either longer or shorter times intervals of cohabitation [73, 77], there is a substantial individual variability in the huddling times in vehicle-injected animals. This variability could be not only due to technical variation of sampling, but also due to biological factors, including genetic polymorphisms regulating regional distribution of relevant receptors [78–81] and epigenetic factors, for example differences in dominance status [82]. In turn, variations in these factors, could contribute to differences in sensitivity and effects of OXTR activation. It would be interesting to investigate the contribution of genetic and epigenetic factors to the effects of OXTR agonists on acquisition and expression of pair bonding.

Since LIT was administered systemically, it could be targeting multiple sites of OXTR expression. Based on previous demonstrations that central sites of OXTR expression are responsible for regulation of social behaviors, it is tempting to hypothesize which of the neurocircuits are involved in signaling the presence of social partner [83]. Earlier studies on viral manipulations of OXTR expression in female prairie voles or pharmacological manipulations of OXTR activity in male prairie voles implicated the nucleus accumbens in promoting pair bonding [32, 34, 75, 76]. Furthermore, chemogenetic inhibition of nucleus accumbens prior to PPT (testing expression of pair bonding) decreased affiliation of female prairie voles with their male partners, while chemogenetic activation of this brain region increased affiliative behaviors towards strangers [84]. Depression-like behavior following viral knockdown of OXTR expression in nucleus accumbens also suggests a potential role of this brain region in expression of other indices of pair bonding [60]. On the other hand, chemogenetic activation of the lateral septum in male prairie voles increased non-specific affiliative behavior and acquisition of pair bonding, but decreased selective aggression, an index of expression of pair bonding [85]. The latter observation suggest that varied brain regions could be involved in regulation of not only different phases of pair bonding but also different behaviors associated with pair bonding. Further assays modulating OXT and OXTR with pathway- and region-specificity will have to be conducted to deduce the exact neurocircuitry that signals the presence of a social partner.

Due to colony limitations, we have tested the effects of OXT agonism only in male animals. The generality of our observations should be also evaluated in female voles. Nevertheless, given the interest in manipulating the OXT system for the treatment of various mental conditions, our observations suggest important translational implications. Specifically, they suggest unpredictable consequences of targeting OXTR for established social affliations which would depend on the history of the interactions and their social conditions. Here we suggest that potential social consequences of administration of OXTR agonist should be kept in mind. These consequences, however, would not preclude the potential benefit to target OXTR in the case of relevant psychiatric conditions.
